# Comparative Analysis of Catabolic and Anabolic Dehydroshikimate Dehydratases for 3,4-DHBA Production in *Escherichia coli*

**DOI:** 10.3390/microorganisms10071357

**Published:** 2022-07-05

**Authors:** Ekaterina A. Shmonova, Ekaterina A. Savrasova, Elizaveta N. Fedorova, Vera G. Doroshenko

**Affiliations:** Ajinomoto-Genetika Research Institute, 117545 Moscow, Russia; ekaterina_savrasova@agri.ru (E.A.S.); elizaveta_fedorova@agri.ru (E.N.F.); vera_doroshenko@agri.ru (V.G.D.)

**Keywords:** microbial production, 3,4-DHBA, protocatechuic acid, dehydroshikimate dehydratase

## Abstract

The production of 3,4-dihydroxybenzoic acid (3,4-DHBA or protocatechuate) is a relevant task owing to 3,4-DHBA’s pharmaceutical properties and its use as a precursor for subsequent synthesis of high value-added chemicals. The microbial production of 3,4-DHBA using dehydroshikimate dehydratase (DSD) (EC: 4.2.1.118) has been demonstrated previously. DSDs from soil-dwelling organisms (where DSD is involved in quinate/shikimate degradation) and from *Bacillus* spp. (synthesizing the 3,4-DHBA-containing siderophore) were compared in terms of the kinetic properties and their ability to produce 3,4-DHBA. Catabolic DSDs from *Corynebacterium glutamicum* (QsuB) and *Neurospora crassa* (Qa-4) had higher K_m_ (1 and 0.6 mM, respectively) and k_cat_ (61 and 220 s^−1^, respectively) than biosynthetic AsbF from *Bacillus thuringiensis* (K_m_~0.04 mM, k_cat_~1 s^−1^). Product inhibition was found to be a crucial factor when choosing DSD for strain development. AsbF was more inhibited by 3,4-DHBA (IC_50_~0.08 mM), and *Escherichia coli* MG1655 Δ*aroE* P*_lacUV5_*-*asbF*_attφ80_ strain provided only 0.2 g/L 3,4-DHBA in test-tube fermentation. Isogenic strains MG1655 Δ*aroE* P*_lacUV5_*-*qsuB*_attφ80_ and MG1655 Δ*aroE* P*_lacUV5_*-*qa-4*_attφ80_ expressing QsuB and Qa-4 with IC_50_ ~0.35 mM and ~0.64 mM, respectively, accumulated 2.7 g/L 3,4-DHBA under the same conditions.

## 1. Introduction

Protocatechuic acid (3,4-DHBA) is a naturally occurring phenolic acid, which is also known as a simple plant secondary metabolite [1]. It possesses antioxidant, antiviral, anti-inflammatory, anticancer, and anti-neurodegenerative activities and can be used in pharmaceuticals, functional foods, and cosmetics [2,3,4,5,6]. Biotechnological production of 3,4-DHBA from renewable sources is promising because 3,4-DHBA is commonly synthesized chemically from petroleum. Moreover, 3,4-DHBA can be transformed into other industrially valuable chemicals for which novel biosynthetic routes have been developed.

**Figure 1:**
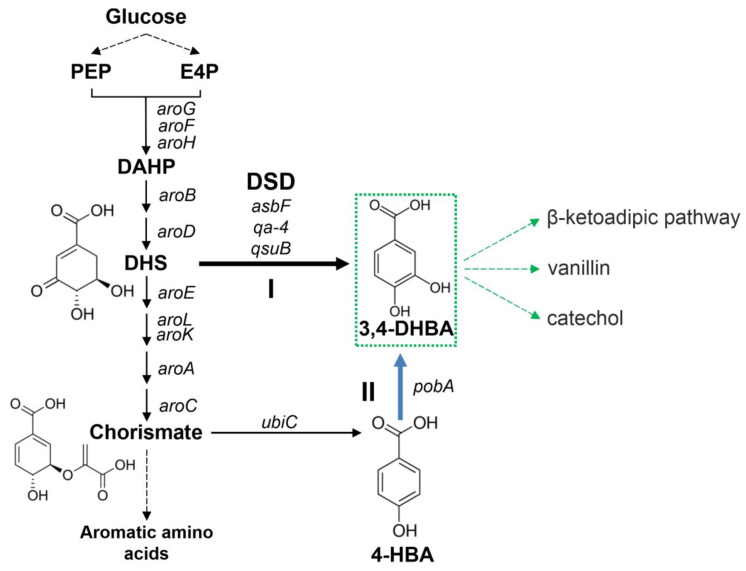
Two routes of 3,4-DHBA biosynthesis (I and II) from glucose. The reactions of common aromatic pathway, which start from condensation of phosphoenolpyruvate (PEP) and erythrose-4-phosphate (E4P) with the formation of 3-deoxy-D-arabino-heptulosonate-7-phosphate (DAHP), are denoted as *E. coli* gene names. *aroG*, *aroF*, *aroH*—DAHP-synthases; *aroB*—3-dehydroquinate synthase; *aroD*—3-dehydroquinate dehydratase; *aroE*—shikimate 5-dehydrogenase; *aroL*, *aroK*—shikimate kinases; *aroA*—5-enolpyruvylshikimate 3-phosphate synthase; *aroC*—chorismate synthase. Bold black and blue lines indicated steps catalyzed by heterologous enzymes. *asbF*, *qsuB*, *qa*-4—DSDs investigated in this work. *ubiC*—chorismate lyase; *pobA*—4-hydroxybenzoate (4-HBA) 3-monooxygenase.

Some microorganisms synthesize 3,4-DHBA as an intermediate of catabolic and anabolic pathways. 3,4-DHBA is formed during the catabolism of quinate/shikimate in soil-dwelling organisms. Then, 3,4-DHBA is shuttled through the β-ketoadipate pathway to produce intermediates of the tricarboxylic acid cycle. In *Bacillus* spp., 3,4-DHBA is a precursor for the production of petrobactin, an iron chelating siderophore. In both cases, 3,4-DHBA is formed from 3-dehydroshikimate (DHS) with the help of DSD. DHS is an intermediate of the common aromatic pathway (Figure 1), and the DSD reaction allows to synthesize 3,4-DHBA from glucose [7] (Figure 1). Another biosynthetic pathway for 3,4-DHBA production was proposed through the end product of the common aromatic pathway chorismate [8] (Figure 1). This biosynthetic route includes more reactions than 3,4-DHBA synthesis via DSD reaction but has other advantages [8].

DSDs are a diverse group of enzymes, which are subdivided into four classes: bacterial single-domain, fungal single-domain, bacterial two-domain, and bacterial membrane-associated enzymes [9]. Bacterial single-domain AsbF from *Bacillus* spp. is an anabolic enzyme that is necessary for the biosynthesis of petrobactin [10,11]. DSDs of other classes belong to the catabolic pathway [9,12]. Membrane-associated DSDs are structurally distinct from the enzymes of the other three classes and exhibit a low level of sequence conservation. The enzymes of the first three classes possess triosephosphate isomerase (TIM) barrel architecture similar to sugar phosphate isomerase [9,11]. Bacterial two-domain DSDs consist of two distinct modules, i.e., an N-terminal isomerase-like domain that is associated with DSD activity and a C-terminal hydroxyphenylpyruvate dioxygenase-like domain that has been proposed to be important for structural stability of the enzyme [9,13].

DSDs derived from different sources have been used for microbial production of 3,4-DHBA and related compounds. Biosynthesis of catechol, vanillin, and the bioplastic precursor muconic acid has been demonstrated in *E. coli* and *Saccharomyces cerevisiae* cells [14,15,16,17,18]. Production of 3,4-DHBA by DSD has been achieved in *E. coli* and *C. glutamicum* cells [13,19,20].

All DSDs used provided 3,4-DHBA levels sufficient for the purposes of each study [13,14,15,16,17,18,19,20]. Thus, different enzymes were not compared with each other to choose the best one. We compared 3,4-DHBA production in *E. coli* cells and kinetic properties of three structurally different DSDs. The results obtained demonstrated a possibility of improving 3,4-DHBA producing strains by selecting an appropriate DSD.

## 2. Materials and Methods

### 2.1. Bacterial Strains, Plasmids, and Growth Conditions

*E. coli* strains were cultivated in LB and SOB media [21]. SOB medium was used for the preparation of electrocompetent cells [22]. Antibiotics were added, when required, in the following concentrations: ampicillin (Ap)—200 mg/L, chloramphenicol (Cm)—20 mg/L, and tetracycline (Tc)—12.5 mg/L.

Cell cultures for protein isolation were prepared as follows. Tubes (18 mm × 200 mm) containing 10 mL of LB with Ap were inoculated with overnight cultures (100 µL) of the BL21(DE3)/pET22b-*DSD* strains and incubated at 25 °C with shaking (200 rpm) for 2 h, then subjected to 1 mM isopropyl β-D-1-thiogalactopyranoside (IPTG) induction and incubated for an additional 20 h.

To determine *E. coli* resistance to 3,4-DHBA (Sigma-Aldrich, St. Louis, MO, USA), an overnight culture of MG1655 strain (LB; 37 °C; 240 rpm) was diluted to OD600 ~0.05 with LB medium supplemented with different 3,4-DHBA concentrations and cultivated in a TVS062CA biophotorecorder (Advantec Toyo Co. Ltd., Tokyo, Japan) at 37 °C (70 rpm).

Test tube (TT) fermentations were prepared in tubes (18 mm × 200 mm) containing 2 mL of the production medium: 40 g/L glucose, 60 g/L CaCO_3_, 10 g/L tryptone, 10 g/L NaCl, 5 g/L yeast extract, 0.5 g/L (NH_4_)_2_SO_4_, 0.5 g/L K_2_HPO_4_, 0.3 g/L MgSO_4_∙7H_2_O, 5 mg/L FeSO_4_∙7H_2_O, 4 mg/L MnSO_4_∙5H_2_O, 10 mg/L thiamine, 10 mg/L 4-hydroxybenzoic acid, 10 mg/L 4-aminobenzoic acid, and 10 mg/L 2,3-dihydroxybenzoic acid. When indicated, IPTG was added (1 mM). The fermentation tubes were inoculated with 0.2 mL of seed culture. To prepare seed culture, one loop (3 mm) of cells from a fresh plate was inoculated into a tube (13 mm × 150 mm) containing 3 mL of LB and incubated at 34 °C with aeration (240 rpm) for 3 h. The fermentation tubes were cultivated at 34 °C (250 rpm) for 44 h. Then, culture broth was diluted to determine OD and product concentrations.

The bacterial strains and plasmids used in this work are shown in Table 1.

### 2.2. DNA Manipulation

DNA manipulation was conducted according to standard procedures [21]. Plasmid DNAs were isolated using Plasmid Miniprep (Evrogen, Moscow, Russia). PCR was performed with Taq DNA Polymerase (GBM, Moscow, Russia) and with Phusion DNA Polymerase (Thermo Scientific, Waltham, MA, USA). Recombinant plasmids were obtained by circular polymerase extension cloning (CPEC) [26]. Primers (Table 2) were purchased from Evrogen (Moscow, Russia). DNA templates for *asbF* and *qa-4* genes were chemically synthesized with codon optimization for *E. coli* (ATG Service Gene, St. Petersburg, Russia). Plasmids and genetic modifications of the *E. coli* chromosome were verified by sequence analysis.

### 2.3. Protein Production and Purification

All manipulations were performed at 4 °C. Cells were harvested after cultivation by centrifugation at 13,200 rpm for 5 min and washed twice with sterile 0.9% NaCl.

Crude extracts were prepared using xTractor™ Buffer (Takara Bio, Mountain View, CA, USA) according to the manufacturer’s instructions.

The supernatants were decanted and then subjected to 12% SDS-PAGE. Protein molecular mass evaluation was performed by comparing with PageRuler Prestained Protein Ladder 26616 (Thermo Scientific, Waltham, MA, USA). Pure hexahistidine-tagged DSDs were isolated using the Capturem™ His-Tagged Purification Miniprep Kit (Takara Bio, Mountain View, CA, USA).

### 2.4. Measurement of DSD Activity

The DSD activities were determined in vitro using the purified C-terminally His-tagged recombinant proteins. The enzymes were incubated in the presence of 1 mM EDTA on ice for 1 h prior to the reactions to remove residual divalent cations. A typical reaction was held in a 1-mL cuvette for 1 min at 20 °C and contained enzyme (150 nM AsbF, 10 nM Qa-4, 20 nM QsuB), 0.1 M Tris/HCl buffer (pH 7.5), 10 mM metal salt, and 0.1–5 mM DHS. The metal cofactors were determined by monitoring the reaction in the presence of 1 mM DHS and each of the tested metal salts: CoCl_2_, MgCl_2_, and MnCl_2_. The inhibition testing was also checked in the presence of 1 mM DHS and 0–0.9 mM 3,4-DHBA addition.

Product identification was performed via comparison of its UV spectrum with that of the 3,4-DHBA standard using a Genesys10S UV-visible spectrophotometer (Thermo Scientific, Madison, WI, USA). The identity of the compounds to DHS and 3,4-DHBA standards was verified using HPLC. For this purpose, ethanol was added to make a concentration of 70% to inactivate the enzyme; the sample was diluted 100-fold in water and filtrated. DHS and 3,4-DHBA standards were purchased from Sigma-Aldrich (St. Louis, MO, USA).

The kinetic properties of the enzymes were measured by following the production of 3,4-DHBA (ε_290_ = 3.89 × 10^3^ M^−1^cm^−1^) at 290 nm using the described procedure [27]. The reaction rate was determined from a linear fit to the change in absorption. V_max_ and K_m_ were obtained by plotting the graph in double reciprocal coordinates.

### 2.5. HPLC Analysis

**Figure 2:**
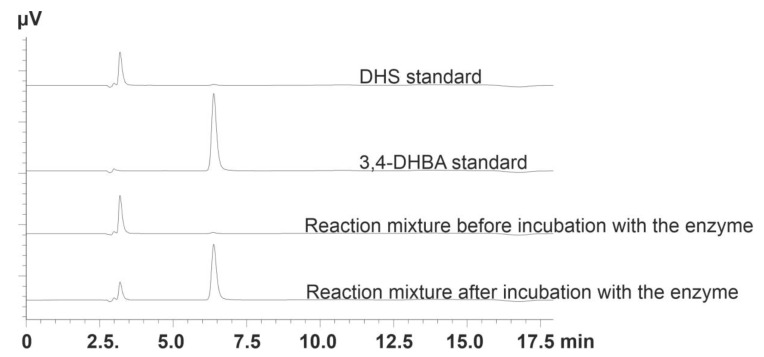
HPLC analysis of the reaction mixtures before and after incubation with the DSDs.

DHS and 3,4-DHBA detection, separation (Figure 2), and concentration determination were performed on a Shimadzu Prominence HPLC system with a diode array detector SPD-M20A (Shimadzu, Maryland, DC, USA) equipped with a Zorbax eclipse column (XDB-c18; 3.0 mm × 150 mm, 3.5 μm) (Agilent Technologies, Santa Clara, CA, USA). Eluent A was 0.025 N H_2_SO_4_, and eluent B was methanol (90%, *v/v*). The methanol gradient varied as follows: 0 min—20%; 7 min—35%; 7–9 min—35%; 10–12 min—50%; 13–18 min—20% at a flow rate of 0.25 mL/min and temperature of 30 °C. UV detection was performed at 235 nm for DHS and 260 nm for 3,4-DHBA.

### 2.6. Sequence Alignment and 3D Structural Analysis

**Figure 3:**
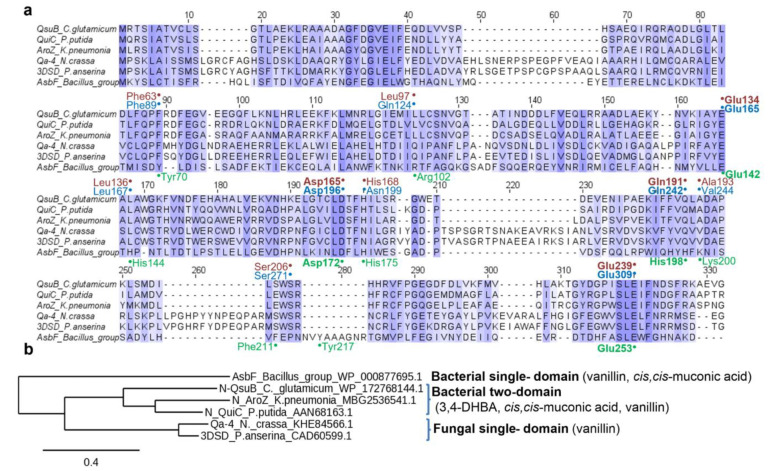
Amino acid alignment (**a**) and a phylogenetic tree (**b**) of DSDs used for the microbial production of 3,4-DHBA and the compounds derived from 3,4-DHBA (noted in brackets). Amino acid residues written in green (below the row), red, and blue (above the row) correspond to AsbF, QsuB, and Qa-4 active center residues, respectively. Bold font indicates residues participating in metal binding.

Multiple sequence alignment of the N-terminal domain from known two-domain DSDs, fungal DSDs, and AsbF was created using T-Coffee software [28]. The corresponding image (i.e., Figure 3a) was generated using Jalview [29]. Phylogenetic analysis was performed using Phylogeny.fr [30]. The 3D structures of QsuB and Qa-4 were predicted using I-TASSER software [31]. Crystal structure of AsbF from *B. anthracis* (PDB ID: 3DX5) was downloaded from Protein Data Bank (http://www.rcsb.org, accessed on 19 February 2022) [32].

### 2.7. Statistical Analysis

All values in graphs and tables are presented as arithmetic means of at least three independent experiments. The given errors are standard deviations. Microsoft Excel 2010 was used for calculations.

## 3. Results

### 3.1. Selection of the Enzymes for Comparative Analysis

Previously, we characterized two-domain QsuB from *C. glutamicum* in terms of its catalytic properties and 3,4-DHBA production in *E. coli* cells [13]. To compare QsuB with structurally different DSDs, the other enzymes used for microbial production were analyzed (Figure 3). They were assigned to three classes.

Bacterial single-domain AsbF was applied for vanillin and muconic acid production in *E. coli* [33,34]. Biochemically and structurally characterized AsbF enzymes from *B. turingiensis* [10] and *B. anthracis* [11] were identical proteins (100% identity). Their amino acid sequences had ~25% identity with the N-terminal domain of two-domain DSDs and ~15% identity to fungal enzymes.

Bacterial two-domain AroZ from *Klebsiella pneumoniae* was used in the pioneering work of Frost’s group to produce 3,4-DHBA, catechol, and vanillin in *E. coli* [14,15]. The prototype of this class of DSDs is QuiC from *P. putida* with a resolved 3D structure (PDB ID: 5HMQ) [9]. The single-domain DSD of *Podospora pauciseta* was applied for vanillin production in yeast [17]. This enzyme was similar (~71% identity) to Qa-4 from other model fungi *N. crassa*, which was the first biochemically characterized DSD [27,35].

We chose AsbF and Qa-4 for a comparative analysis with QsuB.

### 3.2. Overexpression of DSDs in E. coli Using the T7 RNA Polymerase-Based System

**Figure 4:**
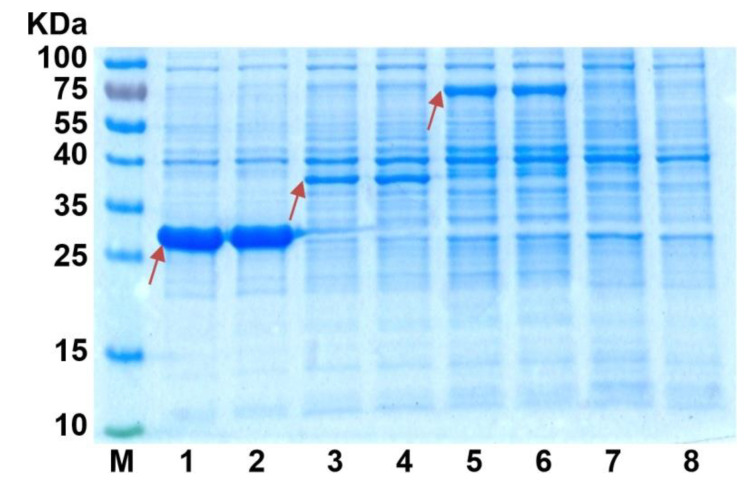
SDS-PAGE of proteins from crude extracts of *E. coli* BL21(DE3)/pET22b-*DSD* cells. Red arrows indicate the target protein. Lane: 1–2—AsbF, 3–4—Qa-4, 5–6—QsuB, 7–8—negative control without DSD. (Overall protein concentration was equal to 10 µg in each lane.)

*asbF* and *qa*-4 genes were cloned in the pET22b vector, and the respective plasmids and previously obtained pET22b-*qsuB* plasmids were used for AsbF, Qa-4, and QsuB overproduction. The analysis of crude extracts of BL21(DE3) cells containing pET22b-*asbF*, pET22b-*qa-4*, and pET22b-*qsuB* plasmids revealed a more intense band for the AsbF protein in comparison with the bands of Qa-4 and QsuB in SDS-PAGE (Figure 4). The elevated expression of the *asbF* gene was explained by the higher efficacy of its hybrid RBS formed from RBS_10_T7 (~25 nucleotides, including the SD sequence of gene 10 from T7 phage and the so-called translational enhancer [36]) present in pET22b plasmid and the 5′-region of the *asbF* coding frame (~35 nucleotides). This conclusion was based on quantitative evaluation of translation efficiency using UTR Designer [37]. This in silico method predicted five- and four-times higher translation of RBS_10_T7-*asbF* versus RBS_10_T7-*qsuB* and RBS_10_T7-*qa-4*, respectively.

AsbF, Qa-4, and QsuB were purified as C-terminally His-tagged recombinant DSDs (Figures S1–S3). The resulting protein samples were used for analysis of DSD activity. Previous activity analysis of purified AsbF and QsuB was also performed on His-tagged recombinant proteins [10,13].

### 3.3. Metal-Dependence Comparison of AsbF, Qa-4, and QsuB Enzymes

**Figure 5:**
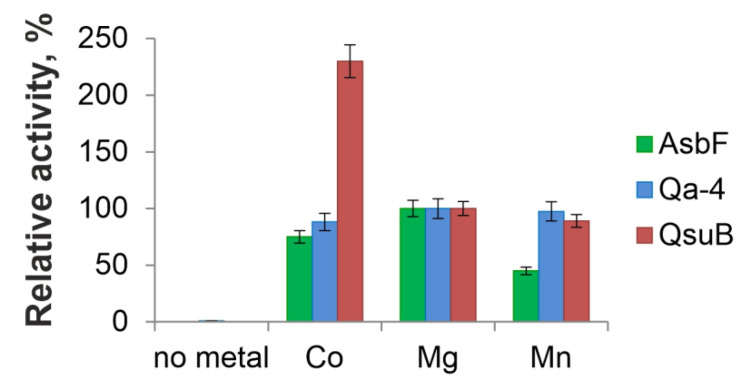
Metal dependency for direct conversion of DHS to 3,4-DHBA by AsbF, Qa-4, and QsuB. Relative DSD activities in the presence of divalent metals were normalized against the activities in the presence of 10 mM MgCl_2_. All activities were tested at physiological pH 7.5 and 20 °C.

DSDs are metal-dependent enzymes capable of using a range of divalent cations [9,10,27]). QsuB provided maximal activity in the presence of Co^2+^ [13]. AsbF from *B. thuringiensis* showed a slight preference for Mg^2+^ over Co^2+^ and Mn^2+^ [10]. Mg^2+^ provided thermal stability for Qa-4, and Mo^2+^, Mn^2+^, Ba^2+^, and Ca^2+^ (preference not shown) restored the enzymatic activity inhibited by EDTA [27]. Taking into account published data, the activities of the isolated proteins were tested in the presence of Co^2+^, Mg^2+^, and Mn^2+^. No activity was observed without addition of metal ions for all proteins (Figure 5). AsbF and Qa-4 were more active with Mg^2+^ ions (Figure 5). The following in vitro experiments were performed with an appropriate metal cofactor for each enzyme.

### 3.4. Comparative Kinetic Analysis of DSDs

**Figure 6:**
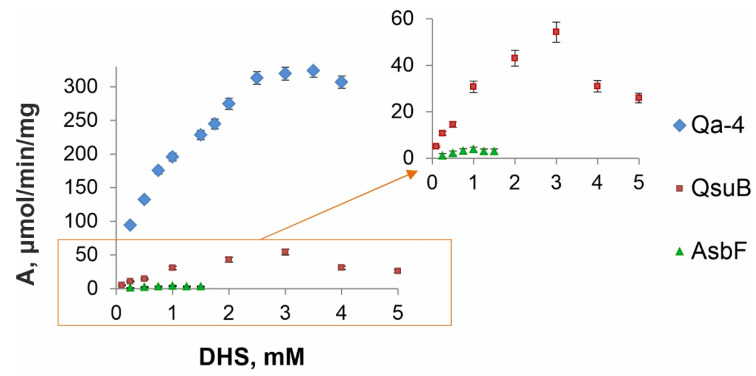
Kinetic curves of DSDs (pH 7.5, 20 °C). The dependence of protein activity on substrate concentration was generated in triplicate. Blue rhombuses represent Qa-4 activity; red squares and green triangles correspond to QsuB and AsbF activities, respectively. The orange frame highlights the area that is magnified in the right graph owing to the considerably higher specific activity of Qa-4 in comparison with that of QsuB and especially with that of AsbF.

It is known that DSD activity is dependent on pH and temperature [10,27,34,35]. We chose physiological pH 7.5 and room temperature (20 °C) for comparison of the enzymes, as previously used for QsuB. These conditions made it possible to evaluate the characteristics of the enzyme, regardless of its thermal stability, and approach the activity of the enzyme in a cell. The activities of the QsuB and Qa-4 enzymes exceeded the activity of AsbF by one and two orders of magnitude, respectively (Figure 6). This affected the calculated enzyme characteristics (Table 3).

The catalytic constant k*_cat_* of Qa-4 was ~220 s^−1^, which was ~3.5 and ~200 times higher than that of QsuB and AsbF, respectively. The substrate specificity of Qa-4 (K*_m_*~600 µM) was significantly worse than that of AsbF (K*_m_*~40 µM), and it was on the same order as that of QsuB (K*_m_*~960 µM). Nevertheless, the catalytic efficiencies (k*_cat_*/K*_m_*) of Qa-4 were 6 and 12 times higher than those of QsuB and AsbF, respectively. According to obtained data, the catabolic enzymes QsuB and Qa-4, overall, were more active than the biosynthetic AsbF enzyme. Obtained parameters were in agreement with data reported previously for Qa-4 (K*_m_* = 5.9 × 10^−4^ M) [27]. In the case of AsbF, our estimations represented intermediate values between K*_m_* of 4.6 ± 1.4 µM and k*_cat_* of 29 ± 2 min^−1^ at 20 °C [34] and K*_m_* of 125 ± 14 µM and k*_cat_* of 217 ± 10 min^−1^ at 37 °C [10].

### 3.5. DSD Inhibition by 3,4-DHBA

**Figure 7:**
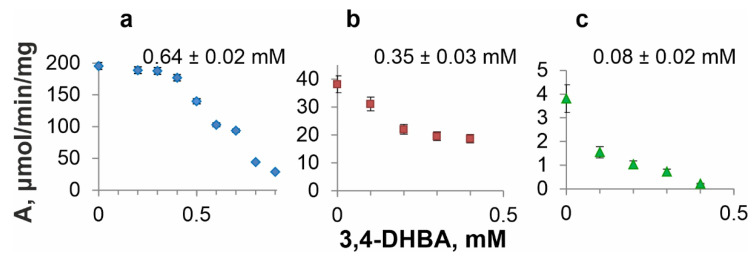
3,4-DHBA inhibition profiles and half-maximal inhibitory constants of DSDs. (**a**) Qa-4, (**b**) QsuB, (**c**) AsbF. IC_50_ values are noted on top of the graphs.

The inhibition of an enzyme by the reaction product is an important characteristic to obtain this product using microbial synthesis. In our previous studies, QsuB inhibition by 3,4-DHBA was investigated, and a noncompetitive inhibition mechanism was established [13]. Thus far, AsbF and Qa-4 have not been characterized in terms of 3,4-DHBA inhibition. However, these enzymes were also found to be inhibited by 3,4-DHBA (Figure 7). For comparison, 3,4-DHBA half-maximal inhibitory constants (IC_50_) of the investigated DSDs were determined (indicated in Figure 7). AsbF was the most sensitive to 3,4-DHBA addition. This enzyme lost more than half of its catalytic activity when 0.1 mM 3,4-DHBA was added. Qa-4 maintained its activity and was practically unchanged up to 0.4 mM 3,4-DHBA (a decrease of <10%). QsuB demonstrated significant loss of activity (~42%) at 0.2 mM 3,4-DHBA.

**Figure 8:**
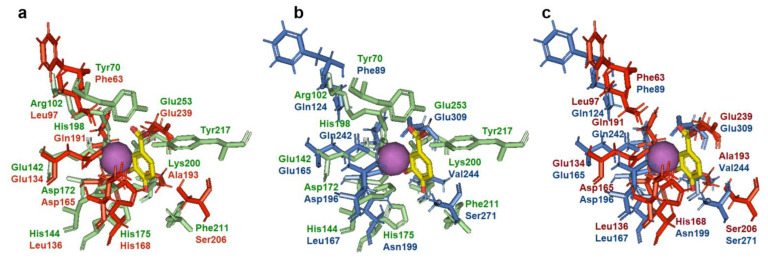
Pairwise active site superimposition of AsbF, QsuB, and Qa-4. 3,4-DHBA and Mn^2+^ were modeled in from the AsbF crystal structure (PDB ID: 3DX5) and is depicted in yellow and as purple sphere, respectively. The residues in green, blue, and red correspond to AsbF, Qa-4, and QsuB, respectively. AsbF crystal structure (PDB ID: 3DX5) and 3D models of QsuB and Qa-4 were used. (**a**) AsbF and QsuB, (**b**) AsbF and Qa-4, (**c**) QsuB and Qa-4.

It is known that 3,4-DHBA is bound to AsbF rather tightly as evidenced by the detection of 3,4-DHBA in the active site of this enzyme [11]. This circumstance made it possible to localize the active/binding center of AsbF. 3D models of QsuB and Qa-4 were created and active centers of AsbF and QsuB, AsbF and Qa-4, and QsuB and Qa-4 were superimposed (Figure 8).

AsbF active center is comprised of the following amino acid residues: Tyr70, Arg102, Glu142, His144, Asp172, His175, His198, Lys200, Phe211, Tyr217, and Glu253. Three residues: Glu142, Asp172, and Glu253 are conserved in all three DSDs (Figure 3 and Figure 8). These residues are responsible for metal ion coordination. QsuB contained additional conserved His198 residue with AsbF. This residue is responsible for 3,4-DHBA binding. Thus, differences in the structure of the active site may contribute to the inhibition of the enzyme by the product. Qa-4 was slightly inhibited by 3,4-DHBA and had no conserved residues with AsbF responsible for 3,4-DHBA binding.

### 3.6. 3,4-DHBA Production Using AsbF, Qa-4, QsuB in E. coli

We have previously studied QsuB for 3,4-DHBA production in *E. coli* [13]. The same approach was used for the comparative characterization of AsbF and Qa-4 enzymes.

*E. coli* did not degrade 3,4-DHBA. Moreover, the MG1655 strain grew in the presence of at least 10 g/L 3,4-DHBA. Therefore, the production of 3,4-DHBA achieved up to 3 g/L using QsuB enzyme was not toxic for host cells and an activation of 3,4-DHBA export was not required. MG1655 ∆*aroE* strain being an aromatic auxotroph accumulated ~3 g/L DHS in a culture broth in the fermentation conditions that was developed earlier.

The genes of DSDs were integrated into the chromosome of the MG1655 ∆*aroE*. Isogenic MG1655 ∆*aroE* P*_laUV5c_*-*asbF*, MG1655 ∆*aroE* P*_lacUV5_*-*qsuB*, and MG1655 ∆*aroE* P*_laUV5c_*-*qa-4* strains were cultivated in TT-fermentation (Table 4). A rich medium was used to support the rapid growth of the strains. After consumption of amino acids, cells stopped growing and glucose consumed was directed to 3,4-DHBA synthesis. IPTG was added for the full induction of DSD genes. The MG1655 ∆*aroE* P*_lacUV5_*-*asbF* strain produced only 0.2 g/L of 3,4-DHBA in the presence of IPTG. MG1655 ∆*aroE* P*_lacUV5_*-*qsuB* and MG1655 ∆*aroE* P*_lacUV5_*-*qa-4* strains accumulated ~2.7 g/L when IPTG was added and ~1 and 2 g/L of 3,4-DHBA without IPTG induction. This was due to promoter leakage expression, which was more pronounced in a strain with more active DSD. All strains also accumulated DHS in amounts inversely proportional to the synthesized 3,4-DHBA.

The low 3,4-DHBA production provided by the MG1655 ∆*aroE* P*_lacUV5_*-*asbF* strain was due to enzyme inhibition by the product but not owing to insufficient gene expression. According to in silico predictions [37], RBS*_lacUV5_*-*asbF* should have been translated 6 and 5 times more than RBS*_lacUV5_*-*qa-4* and RBS*_lacUV5_*-*qsuB*, respectively.

Qa-4, in spite of its better catalytic properties, had no advantages over QsuB in vivo. Strains with QsuB and Qa-4 practically did not accumulate DHS as a by-product. Thus, MG1655 ∆*aroE* cells could be deficient in the precursor of 3,4-DHBA.

## 4. Discussion

For the first time, AsbF, QsuB, and Qa-4 were compared by their catalytic properties in vitro and for a 3,4-DHBA production in vivo. The biochemical characteristics of AsbF obtained in this work correlate with those reported previously in orders of magnitude (Table S1). Qa-4 was previously characterized only by K*_m_*, which coincided with the data of this study (Table S1). IC_50_ values for AsbF and Qa-4 were determined for the first time.

AsbF appeared to be less active and more inhibited by 3,4-DHBA than QsuB and Qa-4. It can be recommended to select catabolic enzymes to synthesize 3,4-DHBA as a final product.

Differences between anabolic and catabolic enzymes are due to their physiological role in a cell. DHS is an intermediate of the common aromatic pathway. Thus, the synthesis of aromatic amino acids and vitamins compete with the 3,4-DHBA synthesis for DHS in microorganisms catabolizing quinate/shikimate as a carbon source. The synthesis of aromatics should have a priority at low DHS concentrations. Indeed, the catabolic DSDs QsuB and Qa-4 had significantly higher K*_m_* values (~1 and 0.6 mM, respectively) compared with shikimate dehydrogenase AroE, which is involved in the synthesis of aromatic compounds. K*_m_* values of AroE were in the range of 0.1–0.2 mM in *E. coli* and *C. glutamicum* [38,39]. In contrast, the synthesis of the 3,4-DHBA-containing siderophore is just as essential as aromatics according to K*_m_* of AsbF for DHS. This enzyme provided 50% activity at approximately 0.1 mM 3,4-DHBA and possessed practically no activity even at 0.4 mM 3,4-DHBA. Nevertheless, AsbF has been successfully used for vanillin and muconic acid production [15,16]. This enzyme is probably more convenient to provide 3,4-DHBA for the next reaction without its accumulation as a by-product.

Qa-4 was more active than QsuB and was less inhibited by 3,4-DHBA. It is likely that the advantages of Qa-4 over QsuB in vivo can be realized in a producer with a higher level of DHS synthesis. MG1655 ∆*aroE* strain used in this study did not contain any modifications enhancing aromatic pathway. At the same time, recent studies showed that catabolic DSDs are highly diverse and it is possible that more advanced enzymes will be found [40,41]

## 5. Conclusions

Three structurally different DSDs (AsbF from *Bacillus* spp., Qa-4 from *N. crassa*, and QsuB from C. *glutamicum*) were compared in vitro and in vivo for 3,4-DHBA production. More active enzymes Qa-4 and QsuB being also less inhibited by 3,4-DHBA are more promising for 3,4-DHBA production in *E. coli* cells.

## Figures and Tables

**Table 1:** *E. coli* strains and plasmids used in this work.

Strain or Plasmid	Relevant Characteristics	Source and Description
*E. coli* strains		
MG1655	Laboratory strain *E. coli* K12 F^−^ λ^−^ *ilvG*^−^ *rfb*-50 *rph*-1	VKPM ^a^ B6195
BL21(DE3)	The strain was used for the expression of genes cloned in the pET22b vector. F–*ompT gal dcm lon hsdSB*(*rB*^–^*mB*^–^) λ(DE3 [*lacI lacUV5-T7p07 ind1 sam7 nin5*]) [*malB*^+^]_K-12_(λ^S^)	Novagen (Merck Millipore, Darmstadt, Germany)
MG1655 ∆*aroE*	The DHS accumulating strain containing in-frame deletion of the *aroE* gene	[13]
MG1655 ∆*aroE* P*_lacUV5_*-*qsuB*_attφ80_	3,4-DHBA producing strains containing DSD genes integrated into the bacterial chromosome and expressed using the IPTG-inducible promoter P*_lacUV5_*	[13]
MG1655 ∆*aroE* P*_lacUV5_*-*asbF*_attφ80_		The integration of P*_lacUV5_*-*asbF* and P*_lacUV5_*-*qa-4* was carried out using “Dual In/Out” method [23] followed by marker excision.
MG1655 ∆*aroE* P*_lacUV5_*-*qa-4*_attφ80_		
Plasmids		
pAH162-λ*attL*-Tc^R^-λ*attR*	The integrative vector for the “Dual In/Out” method	[23]
pELAC	A template for the P*_lacUV5_* promoter amplification, Ap^R^	[24]
pAH123	The helper plasmid containing phage φ80 integrase for the “Dual In/Out” method, Ap^R^	[23]
pMW-*int*-*xis*	The helper plasmid for marker removal: oriR101, repA101ts, λcIts857, λP R → λ*xis*-*int*, Ap^R^	[25]
pET22b	The vector for protein expression, Ap^R^	Novagen (Merck Millipore, Darmstadt, Germany)
pET22b-*qsuB*	The plasmid was used for the production of His-tagged QsuB	[13]
pET22b- *asbF*	The plasmid was used for the production of His-tagged AsbF	The DNA fragment containing pET22b was amplified using the primers P1/P2. The DNA fragments of *asbF* and *qa-4* were amplified using synthetic DNA fragments as templates and the primers P3/P4 and P5/P6, respectively.
pET22b- *qa-4*	The plasmid was used for the production of His-tagged Qa-4	
pAH162-λ*attL*-Tc^R^-λ*attR*- P*_lac_*-*asbF*	The integrative plasmids containing the P*_lacUV5_*-*asbF* modification	The DNA fragment containing P*_lacUV5_*-*DSD* was cloned into pAH162-λ*attL*-Tc^R^-λ*attR* between the *SalI* and *SacI* restriction sites. P*_lacUV5_*-*asbF* and P*_lacUV5_*-*qa-4* were obtained by overlapping PCR (the primers P7/P10 and P7/P12, respectively) of DNA fragments containing the promoter P*_lacUV5_* and *DSD* coding region. These DNA fragments were amplified by PCR with the primers P7/P8 for P*_lacUV5_* and P9/P10 for *asbF*, P11/P12 for *qa-4* using pELAC and pET22b-*DSD* plasmids as templates, respectively.
pAH162-λ*attL*-Tc^R^-λ*attR*-P*_lac_*-*qa-4*	The integrative plasmids containing the P*_lacUV5_*-*qa-4* modification	

^a^ VKPM Russian National Collection of Industrial Microorganisms.

**Table 2:** Primers used in this work.

Primer	Oligonucleotide Sequence
P1	ATGTATATCTCCTTCTTAAAGTTAAACAAAA
P2	CACCACCACCACCACCACTGAGATCCGGCTGCTAACAAAG
P3	TTTTGTTTAACTTTAAGAAGGAGATATACATATGAAATATTCGCTTTGTACTATTAGCT
P4	GTGGTGGTGGTGGTGGTGCGAAGTAACAACTTCCAGTTTGCGA
P5	TTTTGTTTAACTTTAAGAAGGAGATATACATATGCCATCGAAACTAGCAATATCGAGCA
P6	GTGGTGGTGGTGGTGGTGTAACGACGCGGAAACTCGC
P7	TTTTTGTCGACTCTAGAGGATCTGCGGGCAG
P8	ATGTATATCTCCTTCTTAAATCTAGATCCTGTGTGAAATTGTTATCC
P9	TTTAAGAAGGAGATATACATATGAAATATTCG
P10	TCACGAAGTAACAACTTCCAGTTTGCGA
P11	TTTAAGAAGGAGATATACATATGCCATCGAA
P12	TCATGTAACGACGCGGAAACTCGC

**Table 3:** Catalytic properties of DSDs (pH 7.5, 20 °C).

Enzyme	Km (DHS), µM	k*_cat_*, s^−1^	k*_cat_*/K*_m_*, (10^3^ μM^−1^ s^−1^)
Qa-4	598 ± 16	218.6 ± 1.1	365.6 ± 68.8
QsuB	961 ± 77	60.8 ± 0.9	63.3 ± 11.7
AsbF	36 ± 7	1.1 ± 0.1	29.0 ± 7.1

**Table 4:** TT-fermentation from glucose (40 g/L).

MG1655 ∆*aroE S*train	OD_540_	DHS, g/L	3.4-DHBA, g/L	Residual Glucose, g/L	1 mM IPTG
*-*	31 ± 1	3.3 ± 0.1	<0.1	10.0 ± 0.3	+
	31 ± 1	3.4 ± 0.2	<0.1	9.5 ± 0.2	-
P*_lacUV5_*-*asbF*_attφ80_	31 ± 1	2.3 ± 0.1	0.20 ± 0.01	9.5 ± 0.1	+
	30 ± 1	2.3 ± 0.1	<0.1	9.9 ± 0.3	-
P*_lacUV5_*-*qsuB*_attφ80_	30 ± 1	0.2 ± 0.1	2.7 ± 0.2	11.0 ± 1.5	+
	30 ± 1	2.1 ± 0.1	1.0 ± 0.1	10.2 ± 0.2	-
P*_lacUV5_*-*qa-4*_attφ80_	29 ± 1	0.2 ± 0.1	2.7 ± 0.1	12.0 ± 0.8	+
	29 ± 1	0.8 ± 0.1	2.1 ± 0.1	11.0 ± 0.4	-

## Data Availability

The analyzed data presented in this study are included within this article. Further data are available on reasonable request from the corresponding author.

## Supplementary Materials

The following supporting information can be downloaded at: https://www.mdpi.com/article/10.3390/microorganisms10071357/s1, Figure S1: SDS-PAGE of purified AsbF protein; Figure S2: SDS-PAGE of purified Qa-4 protein; Figure S3: SDS-PAGE of purified QsuB protein; Table S1: Analysis of DSD kinetic properties obtained in this work and reported in literature.
