# Supplementary material for: Comparative Analysis of Catabolic and Anabolic Dehydroshikimate Dehydratases for 3,4-DHBA Production in Escherichia coli
Source: Microorganisms. 2022 Jul 5;10(7):1357. doi: 10.3390/microorganisms10071357 (PMC9324987; doi:10.3390/microorganisms10071357)
Supplement: Supplementary file 1 [file microorganisms-10-01357-s001.zip › microorganisms-1764547-supplementary.pdf]

Figure S1. SDS-PAGE of purified AsbF protein.
Lane: 1 – protein molecular mass ladder, 2 – purified AsbF protein (10 µg), 3 – purified AsbF protein (5 µg), 4 – crude extract of *E. coli* BL21(DE3)/pET22b-*asb*F cells (15 µg), 5 – crude extract of *E. coli* BL21(DE3)/pET22b cells (10 µg).

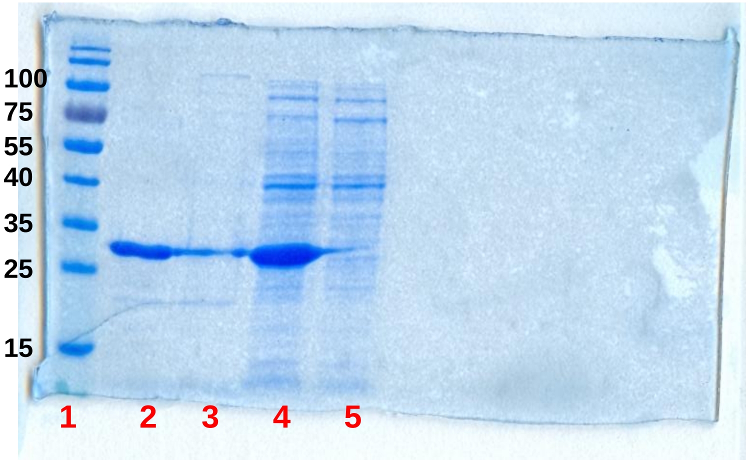

Figure S2. SDS-PAGE of purified Qa-4 protein.

Lane: 1 – protein molecular mass ladder, 2 – crude extract of *E. coli* BL21(DE3)/pET22b-*qa-4* cells (10 µg), 3 – purified Qa-4 protein (10 µg), 4 – *E. coli* BL21(DE3)/pET22b-*qa-4* cells lysate flow through (10 µg).

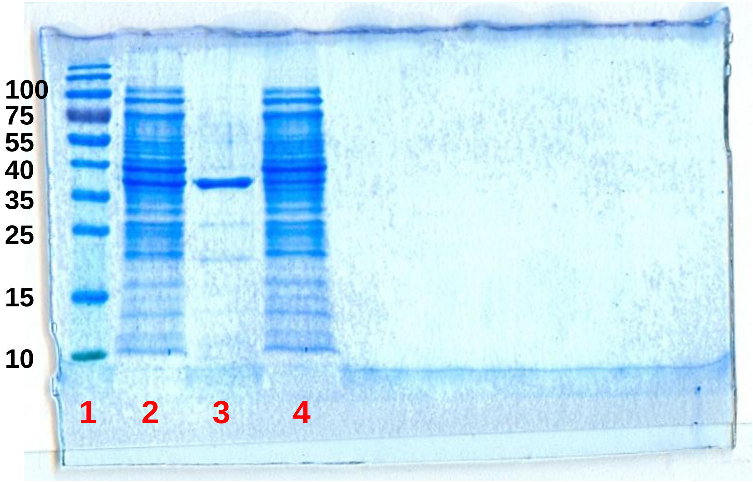

Figure S3. SDS-PAGE of purified QsuB protein.

Lane: 1 – protein molecular mass ladder; 3,5 – crude extracts of *E. coli* BL21(DE3)/pET22b-*qsuB* cells (20 µg); 4,6 – purified QsuB protein (10 µg); x – beyond the scope of current investigation.

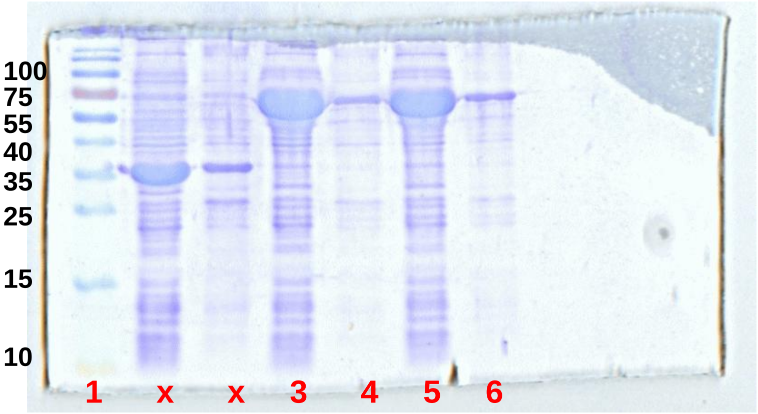

Table S1. Analysis of DSD kinetic properties obtained in this work and reported in literature.

Protein	Source	Kinetic parameters	pH and temperature used	Metal cofactor	Reference
AsbF	*B. anthracis*	K_M 288.7± 38.9μM<br>k_{cat} 79.84 ± 0.96 min^{-1}	pH 7.5 and ambient temperature	Co^{2+}<br>Ca^{2+}<br>Mg^{2+}<br>Mn^{2+} (preferable)	[11]
	B. thuringiensis serovar konkukian 97–27	K_M 2.2 ± 0.4 μM<br>k_{cat} 130 ± 3 min^{-1}	pH 8.6 and 37°C	Mg^{2+}<br>Mn^{2+} (preferable)	[34]
		K_M 4.6 ± 1.4 μM<br>k_{cat} 29 ± 2 min^{-1}	pH 8.6 and 20°C		
		K_M 125 ± 14 μM<br>k_{cat} 217 ± 10 min^{-1}	pH 8.6 and 37°C (opimal pH 8.4-8.8)	Mn^{2+}<br>Co^{2+}<br>Ni^{2+}<br>Mg^{2+} (preferable)	[10]
		K_M 98 ± 11 μM<br>k_{cat} 153 ± 7 min^{-1}	pH 8.6 and 30°C (opimal pH 8.4-8.8)		
		K_M 36 ± 7 μM<br>k_{cat} 63 ± 6 min $^{-1}$	pH 7.5 and 20°C	Mn^{2+}<br>Co^{2+}<br>Mg^{2+} (preferable)	This work
Qa-4	*N. crassa*	K_M 590 μM	pH 7.5 and 37°C	Mn^{2+}<br>Mo^{2+}<br>Co^{2+}<br>Ba^{2+}<br>Mg^{2+}(preferable)	[27]
		K_M 598 ± 16 μM<br>k_{cat} 218.6± 1.1 s^{-1}	pH 7.5 and 20°C	Mn^{2+}<br>Co^{2+}<br>Mg^{2+}(preferable)	This work
QsuB	*C. glutamicum*	K_M 961 ± 77 μM<br>k_{cat} 60.8 ± 0.9 s^{-1}	pH 7.5 and 20°C (opimal pH 8.0-8.4)	Mn^{2+}<br>Mg^{2+}<br>Co^{2+} (preferable)	[13]
QuiC1	*P. putida*	K_M 331 6 ± 51 μM<br>k_{cat} 163.6 ± 8.5 s^{-1}	pH 7.5 and 25°C	Ni^{2+}<br>Mn^{2+}<br>Mg^{2+}<br>Co^{2+}(preferable)	[9]
